# Prevalence of Subjective Cognitive Decline by Social Determinants of Health and Health‐Related Social Needs — 15 States, 2022

**DOI:** 10.1002/alz.087076

**Published:** 2025-01-09

**Authors:** DaJuandra Y Eugene

**Affiliations:** ^1^ Centers for Disease Control and Prevention, Atlanta, GA USA

## Abstract

**Background:**

Dementia is a critical public health issue with known health disparities. Social determinants of health (SDOH) and health‐related social needs (HRSN) may influence differences across various health outcomes, including cognitive functioning. We describe the association between subjective cognitive decline (SCD), an early indicator of possible future dementia, and select adverse SDOH‐HRSN.

**Method:**

We used 2022 data (n = 80,943) from 15 states that administered both the Cognitive Decline and Social Determinants and Health Equity modules in the Behavioral Risk Factor Surveillance System. Prevalence of SCD was estimated among adults aged ≥45 years by 6 adverse SDOH measures (i.e., loss of employment, food stamp use, food insecurity, housing insecurity, inconsistent home utility, lack of transportation) and 3 HRSN measures (i.e., lack of social emotional support, social isolation, stress). Pairwise t‐tests were used to identify significant differences (*P* <.05).

**Result:**

Overall, prevalence of SCD was 11.1% (95% CI: 10.4‐11.8), and SCD was significantly higher among adults reporting each adverse SDOH‐HRSN (e.g., housing insecurity = 27.4%; 95% CI: 23.6‐31.1 vs no housing insecurity = 9.5%; 95% CI: 8.8‐10.1). For adverse SDOH, prevalence of SCD ranged from 19.3% (95% CI: 15.6‐23.1) among those with loss of employment to 32.6% (95% CI: 27.5‐37.8) among those with lack of transportation. For HRSN, prevalence of SCD ranged from 20.3% (95% CI: 18.2‐22.4) among those with lack of social or emotional support to 33.2% (95% CI: 29.7‐36.7) among those with stress.

**Conclusion:**

In 2022, differences in the prevalence of SCD were identified between adults with and without each adverse SDOH‐HRSN examined. Addressing these key drivers of inequities (e.g., transportation), which may lead to barriers in accessing care and services, is essential to help reduce potential disparities in dementia and to support early detection and cognitive health.

